# ZikaPLAN: addressing the knowledge gaps and working towards a research preparedness network in the Americas

**DOI:** 10.1080/16549716.2019.1666566

**Published:** 2019-10-23

**Authors:** Annelies Wilder-Smith, Raman Preet, Elizabeth B. Brickley, Ricardo Arraes de Alencar Ximenes, Demócrito de Barros Miranda-Filho, Celina Maria Turchi Martelli, Thália Velho Barreto de Araújo, Ulisses Ramos Montarroyos, Maria Elisabeth Moreira, Marília Dalva Turchi, Tom Solomon, Bart C. Jacobs, Carlos Pardo Villamizar, Lyda Osorio, Ana Maria Bispo de Filipps, Johan Neyts, Suzanne Kaptein, Ralph Huits, Kevin K. Ariën, Hugh J. Willison, Julia M. Edgar, Susan C. Barnett, Rosanna Peeling, Debi Boeras, Maria G. Guzman, Aravinda M. de Silva, Andrew K. Falconar, Claudia Romero-Vivas, Michael W. Gaunt, Alessandro Sette, Daniela Weiskopf, Louis Lambrechts, Helen Dolk, Joan K. Morris, Ieda M. Orioli, Kathleen M. O’Reilly, Laith Yakob, Joacim Rocklöv, Cristiane Soares, Maria Lúcia Brito Ferreira, Rafael Freitas de Oliveira Franca, Alexander R. Precioso, James Logan, Trudie Lang, Nina Jamieson, Eduardo Massad

**Affiliations:** a Department of Epidemiology and Global Health, Umeå University, Umeå, Sweden; b London School of Hygiene & Tropical Medicine, London, UK; c Departamento de Medicina Tropical, Universidade Federal de Pernambuco, Recife, Brasil; d Departamento de Medicina Interna, Universidade de Pernambuco, Recife, Brasil; e Instituto Aggeu Magalhães, Fundação Oswaldo Cruz, Recife, Brasil; f Departamento de Medicina Social, Universidade Federal de Pernambuco, Recife, Brasil; g Instituto de Ciências Biológicas, Universidade de Pernambuco, Recife, Brasil; h Instituto Fernandes Figueira – Fundação Oswaldo Cruz, Rio de Janeiro, Brasil; i Instituto de Patologia Tropical e Saúde Publica, Universidade Federal de Goiás, Goiânia, Brasil; j Institute of Infection and Global Health, The University of Liverpool, Liverpool, UK; k Departments of Neurology and Immunology, Erasmus Universitair Medisch Centrum Rotterdam, The Netherlands; l Johns Hopkins University, Baltimore, MD, USA; m Universidad del Valle, Colombia; n Laboratório de Flavivírus, Instituto Oswaldo Cruz, Brazil; o Department of Microbiology, Immunology and Transplantation, KU Leuven, Rega Institute of Medical Research, Leuven, Belgium; p Institute of Tropical Medicine, Antwerp, Belgium; q Institute of Infection, Immunity & Inflammation, University of Glasgow, Glasgow, UK; r Insitituto Medicina Tropical, Pedro Kouri, Cuba; s Department of Microbiology and Immunology, University of North Carolina at Chapel Hill, NC, USA; t Departmento del Medicina, Fundacion Universidad del Norte, Barranquilla, Colombia; u Division of Vaccine Discovery, La Jolla Institute for Allergy and Immunology, La Jolla, CA, USA; v Department of Medicine, University of California San Diego, La Jolla, CA, USA; w Insect-Virus Interactions Unit, Institut Pasteur, UMR2000, CNRS, Paris, France; x Maternal Fetal and Infant Research Centre, Institute of Nursing and Health Research, Ulster University, Newtownabbey, UK; y Population Health Research Institute, St George’s, University of London, London, UK; z Associação Técnico-Científica Estudo Colaborativo Latino Americano de Malformações Congênitas (ECLAMC) no Departmento de Genética, Instituto de Biologia, Universidade Federal do Rio de Janeiro, Rio de Janeiro, Brazil; aa Hospital Federal dos Servidores do Estado, Rio de Janeiro, Brazil; bb Hospital da Restauração, Recife, Brazil; cc Oswaldo Cruz Foundation, Recife, Brazil; dd Instituto Butantan, Brazil; ee Pediatrics Department, Medical School of University of Sao Paulo, Sao Paulo, Brazil; ff The Global Health Network, Masters and Scholars of the University of Oxford, Oxford, UK; gg Fundacao de Apoio a Universidade de Sao Paulo, Sao Paulo, Brazil; hh School of Applied Mathematics, Fundacao Getulio Vargas, Rio de Janeiro, Brazil

**Keywords:** Zika, congenital Zika syndrome, birth defect, epidemic preparedness, research capacity building, European Commission, microcephaly, Guillain-Barré syndrome, encephalitis, sustainability

## Abstract

Zika Preparedness Latin American Network (ZikaPLAN) is a research consortium funded by the European Commission to address the research gaps in combating Zika and to establish a sustainable network with research capacity building in the Americas. Here we present a report on ZikaPLAN`s mid-term achievements since its initiation in October 2016 to June 2019, illustrating the research objectives of the 15 work packages ranging from virology, diagnostics, entomology and vector control, modelling to clinical cohort studies in pregnant women and neonates, as well as studies on the neurological complications of Zika infections in adolescents and adults. For example, the Neuroviruses Emerging in the Americas Study (NEAS) has set up more than 10 clinical sites in Colombia. Through the Butantan Phase 3 dengue vaccine trial, we have access to samples of 17,000 subjects in 14 different geographic locations in Brazil. To address the lack of access to clinical samples for diagnostic evaluation, ZikaPLAN set up a network of quality sites with access to well-characterized clinical specimens and capacity for independent evaluations. The International Committee for Congenital Anomaly Surveillance Tools was formed with global representation from regional networks conducting birth defects surveillance. We have collated a comprehensive inventory of resources and tools for birth defects surveillance, and developed an App for low resource regions facilitating the coding and description of all major externally visible congenital anomalies including congenital Zika syndrome. Research Capacity Network (REDe) is a shared and open resource centre where researchers and health workers can access tools, resources and support, enabling better and more research in the region. Addressing the gap in research capacity in LMICs is pivotal in ensuring broad-based systems to be prepared for the next outbreak. Our shared and open research space through REDe will be used to maximize the transfer of research into practice by summarizing the research output and by hosting the tools, resources, guidance and recommendations generated by these studies. Leveraging on the research from this consortium, we are working towards a research preparedness network.

## Background

The European Union funded three research consortia to address the urgent knowledge gaps related to Zika virus (ZIKV), a newly emerged flavivirus that was declared a public health emergency of international concern in 2016 due to its unusual complications [1]. The consortium ‘Zika Preparedness Latin American Network’ (ZikaPLAN) combines 25 multinational and interdisciplinary institutional partners from Europe, Latin America, North America, Africa, and Asia. These 25 institutional partners bring together a full range of expertise ranging from entomology, modelling, birth defect surveillance to clinical studies, coordinated by Umea University, Sweden. All institutions involved in ZikaPLAN, the research design, objectives and overall programme have been described previously in great detail [2]. Table 1 summarizes the work packages and the institutions involved per work package, and their expected impacts. This project was awarded 11.6 million Euro and officially commenced on 1 October 2016, although due to the emergency with a peak in ZIKV cases in early 2016, some of the groups started several research projects before the EU funding arrived. ZikaPLAN is funded for 4 years until 30 September 2020.

**Table 1.** Overview of work packages and their short and long-term impact.

Work Packages	Partners involved in the work package	Addressing urgent gaps and creating the evidence base	Implications for immediate interventions	Long-term research network	Foundation
WP1 MERG	Universidade Federal de Goiás, Universidade Federal de Pernambuco, Umeå University, London School of Hygiene and Tropical Medicine, Associação Técnica–Científica de Estudo Colaborativo Latino Americano de Malformações Congênitas, Fundação Oswaldo Fiocruz and University of Pernambuco.	Determining the attack rate, the case definition of congenital Zika syndrome and the extent of disability and health care impact	Harmonized Latin-American wide guidelines for the management of severe illness caused by Zika	Platform for intervention (vaccine and drug studies)	Research network against any future emerging severe infectious threats
WP2 NEURO-Zika: clinical	Umeå University, University of Glasgow, University of Oxford, Erasmus University, Universidad del Valle, University of Liverpool, Fundação de Apoio à Universidade de São Paulo, and Fundação Oswaldo Fiocruz.	Investigating neurological complications of Zika			
WP3 Non-vector transmission	University of Leuven, Antwerp Institute of Tropical Medicine, Swiss Tropical and Public Health Institute.	Mice models developed to study potential interventions to sexual and vertical transmission; semen studies in ZIKV patients	Potential for additional studies to develop specific therapeutic interventions to mitigate severe complications of Zika infections	Platform for evidence- based public health responses Platform for deployment of large scale pathogenesis studies	
WP4 NEURO-ZIKA: pathogenesis	Umeå University, University of Glasgow, University of Leuven, University of Liverpool, La Jolla Institute for Allergy and Immunology and Fundação Oswaldo Fiocruz.	Pathomechanisms of neuroinvasion and immune mediated response			
WP6 INVADE: Investigating Vaccines in Antibody Dependent Enhancement (ADE)	London School of Hygiene and Tropical Medicine, Fundación Universidad del Norte, La Jolla Institute for Allergy and Immunology and The University of North Carolina at Chapel Hill.	ADE as cause for more severe Zika disease either confirmed or excluded; T and B cell epitopes defined for vaccine and diagnostic applications			
WP5 Platform for diagnostics innovation and evaluation	London School of Hygiene and Tropical Medicine, University, Fondation Mérieux, University of North Carolina at Chapel Hill, Antwerp Institute of Tropical Medicine, Fundação Oswaldo Fiocruz, Instituto de Medicina Tropical Pedro Kourí, Institut Pasteur de Dakar and contributions by Swiss Tropical and Public Health Institute,	Biobank and virtual platform for diagnostics evaluation	Platform for evaluation of diagnostic assays developed within ZikaPLAN and beyond (commercial companies, academia)	Global laboratory platform to evaluate diagnostics for flavivirus infections	
WP7 Contemporary versus historical viral fitness of ZIKV	University of Leuven, Institut Pasteur and Fundación Universidad del Norte.	Determining the phenotypic differences of the current Zika viruses with historical Zika viruses	Designing evidence-informed public health responses	Platform for evidence- based public health responses	
WP8 Disease burden and Risk Assessment	Umeå University, London School of Hygiene and Tropical Medicine, Queen Mary University of London, University of Ulster, Fundação de Apoio à Universidade de São Paulo, Instituto Butantan, Associação Técnica–Científica de Estudo Colaborativo Latino Americano de Malformações Congênitas and Swiss Tropical and Public Health Institute.	Establishing burden of disease and risk of further spread; tools for birth defect surveillance,			
WP9 Mathematical modelling to inform public health policies	Umeå University, London School of Hygiene and Tropical Medicine, University of Oxford, Institut Pasteur, and Fundação de Apoio à Universidade de São Paulo.	Modelling vector control strategies; vaccine strategies; transmission dynamics			
WP10 WEAR: WEarable Aedes Repellant Technologies	London School of Hygiene and Tropical Medicine and Universidad del Valle.	Wash-in detergent formulations and impregnated clothing technologies for the protection against Aedes mosquito bites	Personal protective measures for affected communities	Rapidly scalable intervention	
WP11 PLAN: Preparedness Latin-American Network	Umeå University, London School of Hygiene and Tropical Medicine, University of Oxford, Fondation Mérieux, Fundação Oswaldo Fiocruz, University of Pernambuco, with contributions from all other partners.	Online curriculum and education website, engaging with affected communities, stakeholders and policymakers, adaptable study protocols, resolving regulatory and administrative bottlenecks, coordinating actions within the consortium and other consortia.	Network prepared for rapid support of designing clinical and public health responses	Platform for a Latin American research network for birth defect surveillance, hospital based research, and cohort studies	
WP12 Dissemination and Communication	Fondation Mérieux and Umeå University with contributions from all other partners.				
WP13 Consortium Coordination	Umeå University is coordinator of the consortium.				
WP 14 Zika Data Share	This is a shared WP amongst ZikaPLAN, ZIKAlliance and ZIKAction. Led by the London School of Hygiene and Tropical Medicine for ZikaPLAN.	Enhance outreach through and along the work of the other EU funded consortia, ZIKAction and ZIKAlliance			Network beyond ZikaPLAN
WP 15 ZIKA-COLLAB	This is a shared WP amongst ZikaPLAN, ZIKAlliance and ZIKAction. Led by Umeå University for ZikaPLAN.				

**Figure 1.**
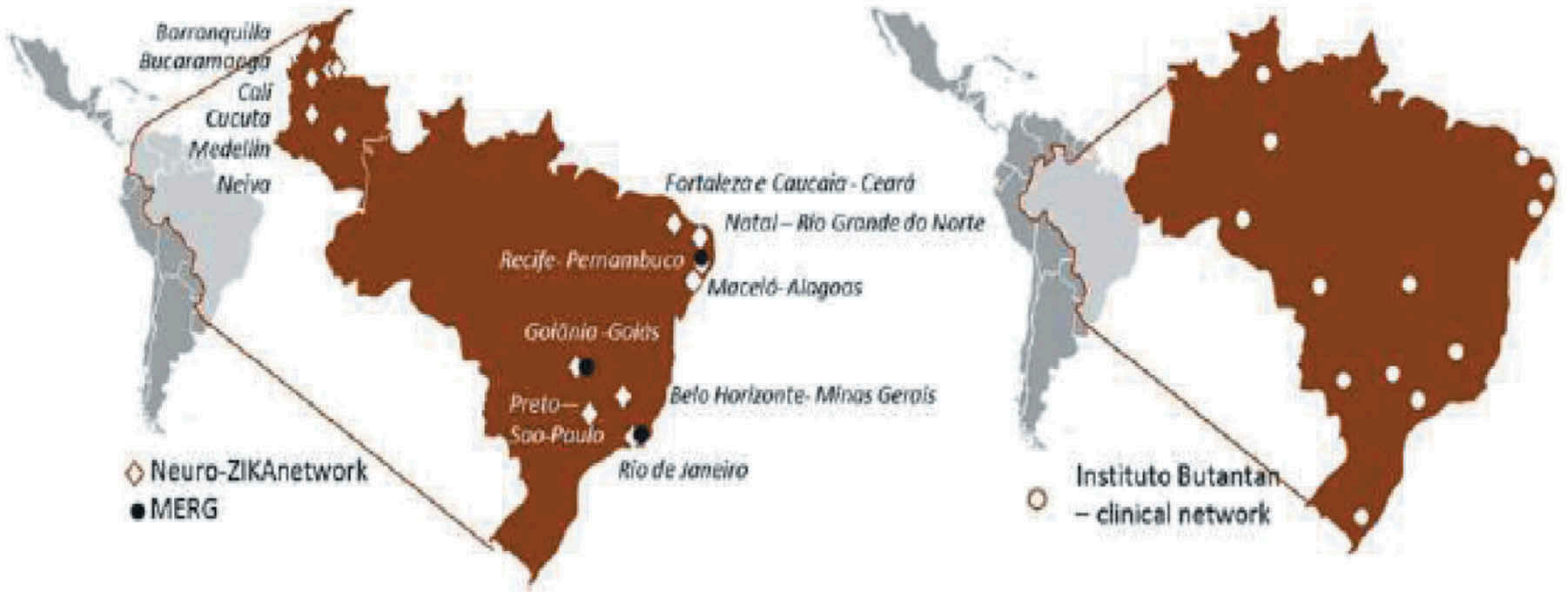
Microcephaly Epidemic Research Group (MERG), Neuro-Zika, and the clinical sites of the Butantan Phase 3 trial for dengue vaccine.

**Figure 2.**
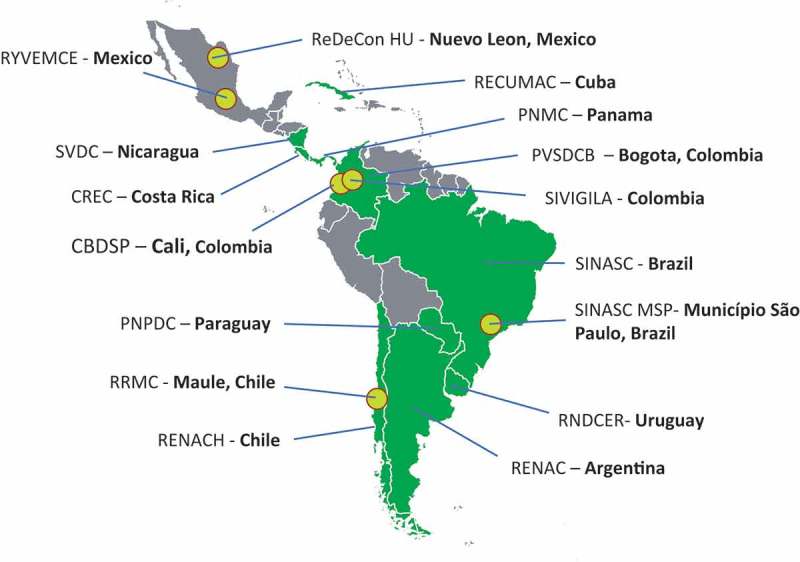
Birth defect surveillance network (RELAMC).

ZikaPLAN is a network of networks, far beyond the 25 beneficiaries as listed in the consortium description [2]. Some of the networks grew out of DengueTools [3], further expanded to include partners from the Latin-American region. Through these networks, we have direct access to various complementary, fully operational (clinical, laboratory and surveillance) networks that are being leveraged in the different studies in ZikaPLAN. These networks will also function as a springboard to the development of the envisaged Latin-American preparedness and response network in the longer term beyond the ZikaPLAN funding period. Here we describe only a selection of such networks, particularly pertinent to ZikaPLAN:

Brazil`s Microcephaly Epidemic Research Group (MERG) was quickly established when the outbreak emerged in North-East Brazil in 2015. It initially focused on case–control studies, established the link between ZIKV and microcephaly, and then expanded its work to set up prospective pregnant women cohort studies [4–8] (Figure 1). To better investigate birth defects, ZikaPLAN partners with ECLAMC, the Latin American Collaborative Study of Congenital Malformations with 21 active participating hospitals in 7 countries in South America (www.eclamc.org) (Figure 2); and EUROCAT, the European surveillance of congenital anomalies (www.eurocat-network.eu/) with 43 registries in 23 countries surveying more than 1.7 million births per year in Europe.

**Figure 3.**
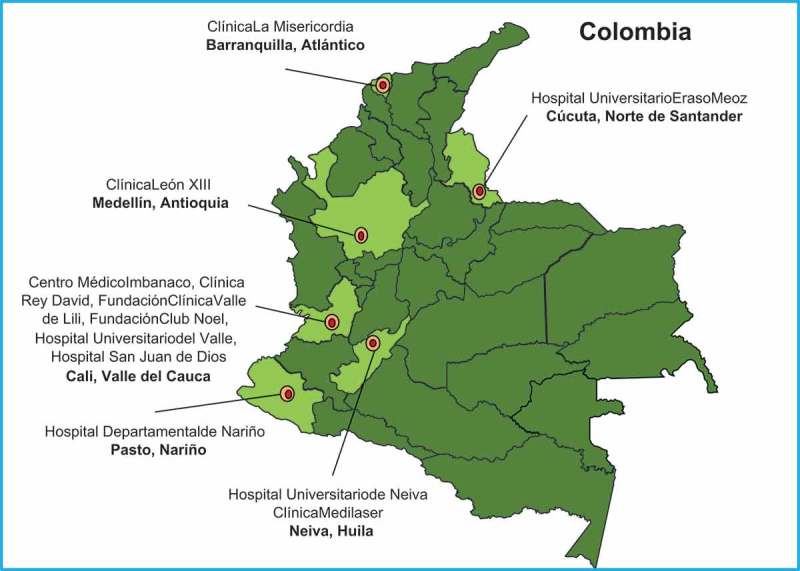
Neuro Emerging Viruses in the Americas Study (NEAS).

The Neuroviruses Emerging in the Americas Study (NEAS) is a collaborative network leading a multi-center study in the Americas to establish a comprehensive registry of the clinical, radiological and laboratory profile of patients with new onset of neurological disorders associated with Zika virus infections (www.neasstudy.org) (Figure 3). Given the links of ZIKV with Guillain-Barre Syndrome (GBS), the International GBS Outcome Study (IGOS) is a particularly important network coordinated by ZikaPLAN partner Erasmus University Medical Center in Rotterdam, The Netherlands [9]. IGOS coordinates clinical data on GBS and biobanks, thereby aiming to identify clinical and biological determinants and predictors of disease course in GBS. IGOS also provides a platform to validate new treatments and to increase the standardization of care in the participating centers.

A pivotal ZikaPLAN partner is the Global Health Network (TGHN) based at Oxford University, UK. TGHN is a knowledge-sharing hub that provides capacity building and process improvement through online platforms, regional and online training, resources and professional development to build skills and careers that deliver evidence to change practice.

Another institutional partner is Butantan. Instituto Butantan is a Brazilian biologic research center located in São Paulo, Brazil. Instituto Butantan is a public institution affiliated with the São Paulo State Secretariat of Health and considered one of the major scientific centers in the world. Butantan is currently running the large Phase 3 trial for a tetravalent live-attenuated dengue vaccine [10] with about 17,000 subjects aged 2 to 59 in 14 locations throughout Brazil. Blood samples will be taken from all trial participants, and Butantan kindly agreed for ZikaPLAN to do a ZIKV seroprevalence study based on a randomly selected sample from those 17,000 subjects (Figure 1).

In addition to the above, to maximize the scientific output and networks, ZikaPLAN collaborates closely with the two other EU-funded consortia called ZIKAlliance (https://zikalliance.tghn.org) and ZIKAction (zikaction.org/).

ZikaPLAN`s 15 work packages were designed to [1] address the urgent knowledge gaps to effectively address the problems of ZIKV and [2] build up a research preparedness network in Latin America and the Caribbean region (Table 1). All publications as a result of ZikaPLAN to date can be found at *https://zikaplan.tghn.org/*.

For the sake of accountability, transparency and timely dissemination, here we provide a report on ZikaPLAN`s mid-term achievements to June 2019, both in terms of addressing the knowledge gaps and initial steps taken to set up a research preparedness network.

(I) **Addressing research gaps**

### Investigating congenital Zika syndrome

Following the emergence of microcephaly in Northeast Brazil in 2015 [11], the causal link between maternal ZIKV infection and birth defects was rapidly confirmed by other groups and by ZikaPLAN`s Microcephaly Epidemic Research Group (MERG) [5,12]. Nevertheless, the absolute risks of miscarriage, stillbirth, microcephaly, and other manifestations of Congenital Zika Syndrome (CZS) in pregnancies with ZIKV infections remained unknown. To address this gap in knowledge, our team of ZikaPLAN-affiliated investigators worked in coordination with the Brazilian Ministry of Health’s ZIKV surveillance programs to initiate prospective cohort studies of pregnant women residing in three of the five regions of Brazil: Recife in the Northeast, Goiânia in the Central-west, and Rio de Janeiro in the Southeast. For the cohort studies, we began recruiting pregnant women with rash mid-epidemic in 2015 and closed enrollment with the decline in transmission in 2017, given the rapid global decline in cases. We tested all pregnant women for ZIKV infections using robust laboratory assays, such as quantitative reverse transcription polymerase-chain reaction (qRT-PCR), Immunoglobulin (Ig) M and IgG3 enzyme-linked immunosorbent assays (ELISAs), and Plaque Reduction Neutralization Tests (PRNT_50_). Our clinical teams prospectively monitored the pregnancies for a range of adverse fetal and birth outcomes.

Analyses based on the pregnancy cohort data are underway to determine the spectrum of risks associated with ZIKV infections in pregnancy and to compare the attack rate of CZS by gestational week of infection and region. In addition to conducting detailed epidemiological analyses within each of the three ZikaPLAN study sites, our team of investigators is also leveraging our data in order to contribute to a series of individual participant data meta-analyses (IPD-MAs). These IPD-MAs include: (i) the Consórcio Coortes Brasil, which is informing ZIKV epidemic preparedness policies in Brazil, (ii) the pooled analysis of results from the EU-funded Zika Consortia, which is facilitating the sharing of best practices and epidemiological methods across the research consortia, and (iii) the WHO-led ZIKV IPD Consortium, which is enabling global pooling of data across all eligible studies [13]. By pooling participant-level data across studies, these IPD-MAs will increase the statistical power for assessing the risks associated with ZIKV infection within potentially important clinical subgroups, such as dengue co-infected pregnant women. Further, by including a diversity of populations, these collaborative data-sharing efforts will allow for meaningful investigations into the sources of heterogeneity in the current estimates of absolute and relative risks of adverse outcomes associated with congenital ZIKV infections. The protocol for the IPD-MA of longitudinal studies of pregnant women and their infants and children has meanwhile been published [13].

Because the ZikaPLAN-affiliated study sites represent some of the first research teams to investigate microcephaly, we are privileged to be able to follow some of the world`s largest cohorts of children with CZS. This valuable work has been supported by the EU funding for ZikaPLAN as well as the Wellcome Trust, the UK Department for International Development, the Medical Research Council, the Brazilian Conselho Nacional de Desenvolvimento Cientifico e Tecnológico, the Secetaria de Vigilancia de Saude, the Fundação do Amparo a Ciência e Tecnologia, and other sources. For this research, children have been recruited from our team’s case–control study [4,14], the prospective pregnancy cohorts, and by clinical referral. In total, our multidisciplinary teams are prospectively following more than 500 children with prenatal ZIKV exposure, approximately half of whom have microcephaly, as well as children who were ZIKV-unexposed. During follow-up visits, a ‘task force’ of clinical specialists, including pediatricians, neurologists, otolaryngologists, speech pathologists, ophthalmologists, infectious disease physicians, dieticians, and physical therapists evaluate the wellbeing of the children and carefully monitor for any physical developmental abnormalities. Children’s neurodevelopment is assessed using the Survey of Well-being in Young Children [15], the General Movement Assessment tool [16], and the Bayley Scales of Infant and Toddler Development, third edition (Bayley-III) [17]. Findings from 94 children without microcephaly who underwent both neuroimaging and Bayley-III testing as part of the Rio de Janeiro-based cohort indicate that the odds of having a Bayley-III score 2 SD below the mean is significantly higher among children with abnormal versus normal brain imaging [18]. Among children with ZIKV-related microcephaly, we have observed functional impairments related to feeding behaviors and a worsening of their nutritional status during the critical first 1000 days of development [19]. Further, our research indicates that, even in the absence of microcephaly, a subset of infants with prenatal ZIKV exposure exhibit impaired motor function with a lower number of age-specific movement patterns than their neurotypical, unexposed peers [20]. Additional analyses are ongoing to describe the full range of CZS-related abnormalities and its evolution in the early life course and across our diverse study settings. The full list of publications associated with the ZikaPLAN cohort studies from the beginning of the ZIKV outbreak until June 2019 is summarized in Table 2.

**Table 2.** Scientific publications by the Microcephaly Epidemic Research Group (MERG) in North-East Brazil and ZikaPLAN Work Package 1 Partners, from 2015 to present.

1. Castanha, P. M. S. *et al*. Perinatal analyses of Zika- and dengue virus-specific neutralizing antibodies: A microcephaly case-control study in an area of high dengue endemicity in Brazil. PLoS Neglected Tropical Diseases, 13:1–17, 2019.
2. Souza, W. V. *et al*. Microcephaly epidemic related to the Zika virus and living conditions in Recife, Northeast Brazil. BMC Public Health, 18:130–136, 2018.
3. Araujo, T. V. B. *et al*. Association between microcephaly, Zika virus infection, and other risk factors in Brazil: final report of a case-control study. Lancet Infectious Diseases, 18:328–336, 2018.
4. Albuquerque, M. F. P. M. *et al*. The microcephaly epidemic and Zika virus: building knowledge in epidemiology. Cadernos de Saúde Pública, 34: 1–13, 2018.
5. Pires, P. *et al*. Neuroimaging findings associated with congenital Zika virus syndrome: case series at the time of first epidemic outbreak in Pernambuco State, Brazil. Childs Nervous System, 34:957–963, 2018.
6. Miranda-Filho, D. B. *et al*. Initial Description of the Presumed Congenital Zika Syndrome. American Journal of Public Health, 106:598–600, 2016.
7. Albuquerque, M. F. P. M. *et al*. Microcephaly in Infants, Pernambuco State, Brazil, 2015. Emerging Infectious Diseases, 22:1090–1093, 2016.
8. Araujo, T. V. B. *et al*. Association between Zika virus infection and microcephaly in Brazil, January to May, 2016: preliminary report of a case-control study. Lancet Infectious Diseases, 16:1356–1363, 2016.
9. Albuquerque, M. F. P. M. *et al*. Pyriproxyfen and the microcephaly epidemic in Brazil – an ecological approach to explore the hypothesis of their association. Memórias do Instituto Oswaldo Cruz, 111:774–776, 2016.
10. Souza, W. V. *et al*. Microcephaly in Pernambuco State, Brazil: epidemiological characteristics and evaluation of the diagnostic accuracy of cutoff points for reporting suspected cases. Cadernos de Saude Publica, 32(4):e00017216, 2016.
11. Leal, M. C. *et al*. Hearing Loss in Infants with Microcephaly and Evidence of Congenital Zika Virus Infection – Brazil, November 2015–May 2016. Morbidity and Mortality Weekly Report, 65(34):917–919, 2016.
12. Franca, R. F. O. *et al*. First International Workshop on Zika Virus Held by Oswaldo Cruz Foundation (Fiocruz) in Northeast Brazil March 2016 – A Meeting Report. PLOS Neglected Tropical Diseases, 10(6):e0004760, 2016.
13. Cordeiro, M. T. *et al*. Results of a Zika Virus (ZIKV) Immunoglobulin M–specific diagnostic assay are highly correlated with detection of neutralizing anti-ZIKV antibodies in neonates with congenital disease. The Journal of Infectious Diseases, 214:1897–1904, 2016.
14. Poretti, A. *et al*. Zika virus infection during pregnancy and small heads: What is the connection and what can be seen by imaging. Atlas of Science. 2016. Avaliable in: <http://atlasofscience.org/zika-virus-infection-during-pregnancy-and-small-heads-what-is-the-connection-and-what-can-be-seen-by-imaging/>
15. Hazin, A. N. *et al*. Computed tomographic findings in microcephaly associated with Zika Virus. The New England Journal of Medicine, 374(22):2193–2195, 2016.
16. Einspieler C, Utsch F, Brasil P, Panvequio Aizawa CY, Peyton C, Hydee Hasue R, et al. Association of Infants Exposed to Prenatal Zika Virus Infection With Their Clinical, Neurologic, and Developmental Status Evaluated via the General Movement Assessment Tool. JAMA Netw Open. 2019;2(1):e187235. Epub 2019/01/19. doi: 10.1001/jamanetworkopen.2018.7235. PubMed PMID: 30657537; PubMed Central PMCID: PMCPMC6431234.
17. Brickley EB, Rodrigues LC. Further pieces of evidence in the Zika virus and microcephaly puzzle. Lancet Child Adolesc Health. 2018;2(3):162–4. Epub 2018/09/01. doi: 10.1016/S2352-4642(18)30021-X. PubMed PMID: 30169250.
18. Lopes Moreira ME, Nielsen-Saines K, Brasil P, Kerin T, Damasceno L, Pone M, et al. Neurodevelopment in Infants Exposed to Zika Virus In Utero. N Engl J Med. 2018;379(24):2377–9. Epub 2018/12/24. doi: 10.1056/NEJMc1800098. PubMed PMID: 30575464.
19. Devakumar D, Bamford A, Ferreira MU, Broad J, Rosch RE, Groce N, et al. Infectious causes of microcephaly: epidemiology, pathogenesis, diagnosis, and management. Lancet Infect Dis. 2018;18(1):e1-e13. Epub 2017/08/29. doi: 10.1016/S1473-3099(17)30398-5. PubMed PMID: 28844634.
20. Lowe R, Barcellos C, Brasil P, Cruz OG, Honorio NA, Kuper H, et al. The Zika Virus Epidemic in Brazil: From Discovery to Future Implications. Int J Environ Res Public Health. 2018;15(1). Epub 2018/01/10. doi: 10.3390/ijerph15010096. PubMed PMID: 29315224; PubMed Central PMCID: PMCPMC5800195.
21. Paixao ES, Teixeira MG, Costa M, Barreto ML, Rodrigues LC. Symptomatic Dengue during Pregnancy and Congenital Neurologic Malformations. Emerg Infect Dis. 2018;24(9):1748–50. Epub 2018/08/21. doi: 10.3201/eid2409.170361. PubMed PMID: 30124410; PubMed Central PMCID: PMCPMC6106414.
22. Paixao ES, Leong WY, Rodrigues LC, Wilder-Smith A. Asymptomatic Prenatal Zika Virus Infection and Congenital Zika Syndrome. Open Forum Infect Dis. 2018;5(4):ofy073. Epub 2018/05/08. doi: 10.1093/ofid/ofy073. PubMed PMID: 29732381; PubMed Central PMCID: PMCPMC5925427.
23. Paixao ES, Teixeira MG, Rodrigues LC. Zika, chikungunya and dengue: the causes and threats of new and re-emerging arboviral diseases. BMJ Glob Health. 2018;3(Suppl 1):e000530. Epub 2018/02/13. doi: 10.1136/bmjgh-2017-000530. PubMed PMID: 29435366; PubMed Central PMCID: PMCPMC5759716.
24. Paixao ES, Costa M, Teixeira MG, Harron K, de Almeida MF, Barreto ML, et al. Symptomatic dengue infection during pregnancy and the risk of stillbirth in Brazil, 2006–12: a matched case-control study. Lancet Infect Dis. 2017;17(9):957–64. Epub 2017/08/29. doi: 10.1016/S1473-3099(17)30366-3. PubMed PMID: 28845800; PubMed Central PMCID: PMCPMC6205624.
25. Rodrigues LC, Paixao ES. Risk of Zika-related microcephaly: stable or variable? Lancet. 2017;390(10097):824–6. Epub 2017/06/26. doi: 10.1016/S0140-6736(17)31478-2. PubMed PMID: 28647174.
26. Rodrigues LC. Zika’s Long Haul: Tackling the Causes of Human Vulnerability to Mosquito-Borne Viruses. Am J Public Health. 2017;107(6):831–3. Epub 2017/05/13. doi: 10.2105/AJPH.2017.303792. PubMed PMID: 28498761.
27. Wilder-Smith A, Wei Y, Araújo TVB, VanKerkhove M, Turchi Martelli CM, Turchi MD, et al. Understanding the relation between Zika virus infection during pregnancy and adverse fetal, infant and child outcomes: a protocol for a systematic review and individual participant data meta-analysis of longitudinal studies of pregnant women and their infants and children. BMJ Open. 18 June 2019;9(6):e026092. doi: 10.1136/bmjopen-2018-026092
28. Moreira MCN, Nascimento M, Mendes CHF, Pinto M, Valongueiro S, Moreira MEL, Lyra TM, Kuper H; SEIZ Research Group. Emergency and permanence of the Zika virus epidemic: an agenda connecting research and policy. Cad Saude Publica. 2018;34(8):e00075718.
29. Kuper H, Lyra TM, Moreira, MEL, Albuquerque MSV, Araujo, TVB, Fernandes, S, Jofre-Bonet, M, Larson, H, de Melo, APL, do Nascimento, MAF, Penn-Kekana, L, Pimentel, C, Pinto, M, Simas, C, Valongueiro, S. Social and economic impacts of congenital Zika syndrome in Brazil: Study protocol and rationale for a mixed-methods study [version 2; peer review: 1 approved, 1 approved with reservations] Wellcome Open Research. 2019;3:127

Support for affected communities, in particular, access to health care for children with CZS and their families is a priority, and to this end, ZikaPLAN partners are working towards family support programmes [21,22].

The Zika epidemic in Latin America has brought the world’s attention to the need for effective congenital anomaly (birth defect) surveillance systems. The Birth Defect Surveillance Group within ZikaPLAN is investigating the epidemiology of CZS and is aiming to strengthen birth defects surveillance in Latin America and other low resource regions of the world. We formed the International Committee for Congenital Anomaly Surveillance Tools with global representation from regional networks conducting birth defects surveillance. This committee has collated a comprehensive inventory of resources and tools for birth defects surveillance available on the newly launched global birth defects website (https://globalbirthdefects.tghn.org). An App for low resource regions facilitating the coding and description of all major externally visible congenital anomalies including microcephaly and CZS will begin field-testing in March 2019. Furthermore, we launched the Red Latino Americana de Malformaciones Congénitas (RELAMC), a network of population-based congenital anomaly registries, in November 2018. Data from RELAMC on microcephaly in the pre-ZIKV period have been analysed to provide a baseline [23]. PAHO and RELAMC registry data on microcephaly and CZS as well as any information on congenital maternal infection syndromes in Latin America are currently being analysed.

### Studying the neurological complications of Zika

The full spectrum of neurological disease associated with ZIKV, beyond congenital disease, is poorly understood, and case definitions are further complicated by the fact that other arboviruses such as dengue and chikungunya viruses often co-circulate causing similar disease. The frequency of these neurological manifestations is unknown, although, for Guillain-Barré syndrome (GBS) after Zika, it is estimated to be 2.0 [95% credible interval 0.5–4.5] per 10,000 cases of Zika infection [24]. To address this, we established a Zika Neurology Network (ZNN) in Brazil and retrospectively studied neurological disease cases associated with ZIKV in Rio de Janeiro from November 2015 to June 2016. We found that ZIKV was associated with a wide range of central and peripheral nervous system manifestations, including meningoencephalitis, myelitis, acute disseminated encephalomyelitis, radiculitis and GBS; chikungunya virus appeared to have an equally important association with neurological disease in Brazil, and many patients had dual infection [25]. To investigate this further, we set up parallel case–control studies in Brazil through the ZNN and in Colombia with the NEAS network, recruiting patients presenting with new onset of a neurological syndrome of unknown etiology, including but not limited to encephalitis, meningitis, myelitis, GBS and cranial nerve disease, plus controls. By June 2019, 137 cases with GBS, 245 cases of CNS or cranial nerve inflammatory disorders and 715 controls have been recruited in Brazil, whilst 126 patients with GBS, 64 with CNS inflammatory disorders, and 93 controls have been recruited in Colombia. Analyses are underway, and we expect that the relative proportion of ZIKV as a cause for neurological disease has declined over time; nevertheless, understanding the role of other arboviral diseases in the pathomechanism of neurological disease is equally important so that we continue to build up this project despite the disappearance of ZIKV as a public health problem.

The unusual clusters of ZIKV associated GBS were first described by other groups in French Polynesia [26]. We expanded the already existing IGOS, the largest global observational cohort study on GBS [9], to establish a case–control study for Zika (IGOS-Zika), that is now running in nine hospitals across three countries (Brazil, Argentina, Malaysia). International data and sample collection were further standardized by sharing the IGOS protocol with networks in Latin America (NEAS, CDC, Zika Neurology Network) [27]. The IGOS database will also facilitate the comparison between ZIKV-related GBS and GBS after other infections. Additionally, collaborations with local neurologists in Recife, Brazil, are ongoing to describe a GBS cohort collected early in the ZIKV outbreak. As clinical guidelines for the management of GBS are currently lacking [28], we developed a globally applicable consensus report in collaboration with international and local experts to support the diagnosis and management of GBS. This guideline, as well as results from an ongoing survey amongst Brazilian neurologists, will be used to develop training material and courses in collaboration with The Global Health Network.

It is important to discover and characterise the mechanistic pathways of ZIKV infection in the pathogenesis of central (CNS) and peripheral nervous system (PNS) injury, focusing on both direct viral invasion, and the immune and autoimmune responses to viral infection. ZIKV tropism in the peripheral and central nervous systems (PNS and CNS, respectively) has now been defined *in vitro* and these data are guiding progress through *in vivo* work in which a neurological phenotype is also seen. We found the resistance of Schwann cells to infection, compared with CNS cells [29]. Different viral isolates show different tropisms for nervous system cells. We are using infection of induced pluripotent neural stem cells and other neural cells to conduct drug screens. Identification of ZIKV main cellular receptors on human neural cells is ongoing with unbiased and candidate approaches. Studies using sera from Zika-GBS cases and controls have been screened for known GBS antigen targets, but to date, no antigen targets have been found indicating the ZIKV associated GBS may have a unique autoimmune signature. We have now expanded this work to include different *in vitro* culture systems. The cellular studies to examine differential immune responses in patients with non-neurological ZIKV infection, patients with viral invasion of the CNS (e.g. encephalitis) and patients with autoimmune neurological disease are awaiting the collection of carefully archived clinical samples obtained from ZikaPLAN`s studies in various hospitals in Brazil and Colombia.

### Developing diagnostics for Zika

Lack of suitable diagnostic assays has hampered Zika research and the public health emergency response [30]. Novel diagnostics often do not make it to the finish line due to lack of access to specimens needed and need to be validated before use in a clinical setting [31]. To address these barriers, ZikaPLAN set up a network to accelerate the development and evaluation of Zika diagnostics for clinical and surveillance use. To this end, a network of quality sites with access to well-characterized clinical specimens and capacity for independent evaluations were selected based on GCP/GCLP criteria. Specimens from confirmed Zika positive and negative patients as well as from dengue confirmed patients are being collected during the acute and convalescent stage. Sites adhere to the same protocol standards and compare new diagnostics to reference standards. A network of sites provides the framework for a global biobank and quality laboratories for multi-site evaluations with rapid data collection, analysis and reporting. Standardized panels are representative of different geographic areas. Two of these commercial assays have been evaluated at three study sites in Colombia, Senegal and Thailand, and which were designed to detect patients’ specific anti-ZIKV IgG and IgM antibodies. Additional four or more assays will be evaluated in 2019. While the results are still pending, this approach reduced the time for test evaluations and hence can resolve the bottleneck for market entry of ZIKV diagnostics.

To enhance diagnostics development, ZikaPLAN has been actively involved in characterizing humoral and T cell responses. Through comparisons of known epitope sequences in their native structural conformations on target proteins from other flaviviruses with the corresponding sequences of ZIKV, candidate-conserved and cross-reactive ZIKV B-cell epitopes were identified on the E and NS1 proteins, while these ZIKV T cell epitopes were identified in the NS3 and NS5 protein sequences [32]. Patients who encountered sequential DENV and ZIKV infections generated much stronger CD4+ and CD8 + T-cell responses during the subsequent ZIKV infection due to the presence of common epitopes on these viruses, despite the major target proteins for T cell responses on ZIKV being different from those on the DENVs [33]. The ontogeny of the B-cell and T-cell responses of a ZIKV infected, but DENV-naïve, patient were also fully assessed [34]. In a more recent study, we showed that CD8 + T-cells generated against ZIKV to exhibit an upregulated immune activation, cytokine production and target cell cytotoxicity gene profiles [35].

ZikaPLAN researchers also analysed the molecular specificity of the human antibody response to ZIKV. Using human monoclonal antibodies isolated from Zika patients, type-specific epitopes on domains I, II and III of the ZIKV envelope (E) protein were defined [36]. Recombinant antigens displaying type-specific epitopes were useful for the sero-diagnosis of ZIKV infections [37]. These antigens have been used to understand the sero-epidemiology of ZIKV in Asia and Latin America [38]. New diagnostic antigens have been shared with collaborators in academia and industry to support the development of rapid diagnostic tests.

To date, we have prepared various chimeric constructs encoding the pre-membrane (prM) encoding genes of either the African or French Polynesian/South American strains with those encoding the main envelope (E) for expression as recombinant viral-like particles in mammalian and insect cells. Importantly, these constructs will be used in functional (cell-binding and antibody-dependent enhancement (ADE) assays to account for the ZIKV strain variations observed from previous sequence analysis) [39].

The antigenic sites implicated in the generation of a dramatic and lethal dengue virus ADE in outbred mice using polyclonal and monoclonal antibodies [40] are now also being tested against ZIKV. Recombinant subunit antigens and synthetic peptide preparations, based on their protein x-ray diffraction structural determinations or computer predictions, are also being prepared to assess their diagnostic, ADE and pathogenic capacities. The first of our studies on ZIKV CD8 + T-cell epitope analysis together with their gene expression profiles were performed and published and they also showed these ZIKV responses were robust and not affected by geographical location, time after ZIKV infection or previous DENV infections [35]. Chimeric constructions of different (e.g. African, Asia/Pacific and American) ZIKV strains have been prepared by our team as virus-like particles (VLPs) to confirm their roles in pathogenesis by target cell-binding and ADE.

### Exploring reasons for the explosive emergence of ZIKV

The factors that have fueled the explosiveness and magnitude of ZIKV emergence in the Pacific and the Americas are still poorly understood. Reciprocally, the lack of major human epidemics of ZIKV in regions with seemingly favorable conditions, such as Africa or Asia, remains largely unexplained [41]. The potential contribution of vector population diversity to ZIKV epidemiological patterns may have been overlooked. To address this question, we established dose–response curves for eight field-derived populations of the major vector *Aedes aegypti* representing the global range of the species, following experimental exposure to six low-passage ZIKV strains spanning the current viral genetic diversity. Our results revealed that African *Ae. aegypti* are significantly less competent than non-African *Ae. aegypti* across all viral strains tested. This finding suggests that low vector competence may have contributed to preventing large-scale human transmission of Zika virus in Africa (manuscript in preparation).

Tracing the phylogenetic history and spatio-temporal dispersal pattern of ZIKV in Asia prior to its explosive emergence in the Pacific and the Americas, there was an extended period of relatively silent transmission in South-East Asia, enabling the virus to expand geographically and evolve adaptively before its unanticipated introduction to immunologically naive populations on the Pacific islands and in the Americas, as reported by other groups [41]. Substitutions in ZIKV may be implicated in strain variation in the emergence of ZIKV strains from Africa into South and South-east Asia [41]. The amino acid substitutions, possibly implicated in the neurological pathogenesis and possible antibody-dependent enhancement (ADE) of severe ZIKV strains that emerged in French Polynesia and subsequently swept through Latin America, were also identified [39].

To explore whether there is a differential viral fitness between contemporary ZIKV strains versus historic ZIKV strains, we are in the process of studying histopathological changes as well as viral load in brain, spinal cord and testicles in mice. Various mouse species were first evaluated for their susceptibility to ZIKV infection. Both immunocompromised (AG129 and Ifnar^−/-^) and immunocompetent (C57BL/6 and BALB/c) mice were inoculated with the ZIKV MR766 prototype strain, obtained from the European Virus Archive (EVA) (http://www.european-virus-archive.com). Since immunocompetent mice are generally resistant to infection with flaviviruses, immunocompetent mice were treated with an interferon (IFN) receptor-blocking antibody prior to infection to render them more susceptible to ZIKV replication. In contrast, both AG129 mice and Ifnar^−/-^ mice are highly susceptible to flavivirus infections because these mice lack receptors for IFN-α/β (Ifnar^−/-^) as well as IFN-γ (INF-α/β/γR-/-/-: AG129).

We showed that ZIKV replicated well in all mice except in C57BL/6, where half of the mice were non-responsive to ZIKV infection. Next, virus-induced disease brought about by a historical and a contemporary ZIKV strain, the MR766 strain from EVA and the SL1602 isolate from Suriname [42], respectively, was evaluated only in AG129 mice. Inoculation with the SL1602 strain resulted in a faster progression to disease than was the case with MR766 (manuscript in preparation). Altogether, these studies demonstrate that AG129 mice are suitable to study the viral fitness of contemporary versus historic ZIKV strains.

In humans, several other research groups have shown that ZIKV RNA and infectious virus have been detected in semen up to 6 months and 3 months post infection, respectively [43]. In the initial phase of the epidemic, there were concerns that sexual transmission may increase the risk of infection and epidemic size. These concerns have been abated using mathematical modeling by our group [44], which illustrated that for sexual transmission to be a stand-alone epidemic threat, the duration of infectiousness would need to be beyond biologically plausible values.

### Investigating the evolution of the Zika epidemic

Analyses of viral genomes with ecological and epidemiological data yield an estimate that ZIKV was present in Northeast Brazil by February 2014 and is likely to have disseminated from there, nationally and internationally, before the first detection of ZIKV in the Americas [45]. This was corroborated by a ZikaPLAN`s modeling study [46]. Collating information on confirmed and suspected Zika cases across Latin-American countries [https://github.com/kath-o-reilly/Zika-LAC-Outbreaks] a spatio-temporal dynamic transmission model for ZIKV infection was used to project its incidence in 90 major cities within 35 countries. A key output from the model included the clear demonstration that the ZIKV epidemic predominantly finished before 2018, with potential for only small pockets of low numbers of infections occurring in the region since [47]. Fitting to, and validation with, region-wide incidence data from the complete epidemic has resulted in unparalleled robustness in model parameterisation, allowing for new insights into the spatial evolution of the epidemic. Unusually, flight data seemed to provide a poor fit to the region-wide spread of infection. Instead, Zika’s spread was best explained by a model of infection that followed land travel to nearby large cities [47]. Our modelling findings confirm that the ZIKV epidemic is by and large over within Latin America. Local low levels of transmission are probable, but the estimated rate of infection suggests that most cities in Latin America have a population with high levels of herd immunity. Of note, ZIKV continues to circulate at a low level in Asia [48] and Africa, with small clusters of outbreaks in India and Angola [49,50].

Predictions on further ZIKV epidemiology also have implications on the use of potential ZIKV vaccines. WHO has developed a target product profile for a ZIKV vaccine for outbreak use. ZikaPLAN presents a model to optimize a vaccination campaign aiming to prevent or to curb a Zika virus outbreak. We show that the optimum vaccination strategy to reduce the number of cases by a mass vaccination campaign should start when infected *Aedes* mosquitoes reach a density of greater than 1.5 mosquitoes per humans [51].

Lastly, thanks to the access to Butantan`s blood samples in the context of a Phase 3 dengue vaccine trial among 17,000 subjects in Brazil, we are currently also in the process of conducting a seroprevalence study for Zika, stratified by age, gender, geographic location and prior dengue exposure to better understand the evolution of Zika in Brazil.

### Exploring the role of travel and travellers

The explosive spread of Zika virus in the Western hemisphere has been attributed to the ever-increasing volume of international travellers to countries with vector-borne transmission [52]. Consequently, many travellers were affected during the outbreak [53–59], and travellers served as sentinel to unmask Zika transmission in countries beyond the Americas [53,56,59,60]. Graded evidence for best practices [61,62] will be needed for travellers, and sentinel surveillance in returning travellers is one important tool to study the burden, evolution over time and spectrum of disease in travellers. ZikaPLAN is working with two networks that offer sentinel surveillance. GeoSentinel is the largest sentinel network in returning travellers [63], which allows to investigate the effect of purpose or sub-population of travel [64,65], destination [66,67], as well as disease-specific questions [68–70]. Through GeoSentinel, we are currently exploring the geographic spread of Zika in travellers globally over time. Furthermore, expanding from our previous collaboration with the European network on sentinel surveillance in returning travellers (TropNet) through DengueTools [71], we prospectively enrolled travellers with laboratory-confirmed ZIKV infections. We found the incidence rate of ZIKV infection in European travellers to affected territories to be as high as 17% (95% confidence interval, 8 to 32) per month of travel during the height of the outbreak [72]. In this study, all symptomatic travellers had been infected within 3 weeks of arrival in areas that reported ZIKV circulation. Asymptomatic ZIKV infection was rare in this population without prior exposure to *flavivirus* infections. We calculated that ZIKV-infection can be safely ruled out when negative results are obtained in an NS1-based ZIKV-antibody detection assay, performed at 20–24 days post symptom onset or last possible exposure [73]. Virus shedding in the semen is close to zero for dengue [74,75] but in line with studies from endemic areas [43], ZikaPLAN found that 60% of male ZIKV cases among returning travellers had detectable ZIKV RNA in their semen for a median duration of 83 days [76]. These findings led to the pre-conception guidance for travellers [77]. With the rapid global decline of ZIKV infections, we discontinued the studies in travellers in 2017, and are now focusing on mosquito trap studies distributed via international travellers.

Furthermore, ZikaPLAN is working with BlueDot which has access to air passenger data through International Air Transport Association (IATA) on various modelling exercises to determine the spread of Zika and other viruses via transportation networks in particular via air travel [46,49,78–80].

### Addressing best practices for vector control

By transmitting dengue, chikungunya and yellow fever viruses as well as ZIKV, *Aedes* (*Ae*.) *aegypti* mosquitoes exert a huge toll on global health. This mosquito species is highly invasive, continually expanding its global distribution each year [81]. Despite significant investments, the incidence of arboviral infections continues to show a steady upward trend. Novel arbovirus vector control tools are desperately needed and several alternative approaches are currently under development with preliminary trials underway to meet this need [82]. Two novel approaches that have shown considerable promise in field trials in recent years are the development of mosquitoes that are resistant to arboviral infection and the genetic control of *Ae. aegypti* mosquitoes. Transinfection of mosquitoes with *Wolbachia*, a naturally occurring endosymbiotic bacterium, reduces mosquito infection with all of these arboviruses [83]. Genetic control using a technology known as RIDL (the Release of Insects carrying Dominant Lethal genes) gives rise to non-viable offspring thereby suppressing the mosquito populations [83,84]. ZikaPLAN`s modelling research has highlighted that these technologies used together can give rise to phenomenal synergisms, facilitating both the suppression of the wild mosquito vectors and the spread of their arbovirus-resistant (*Wolbachia*-carrying) replacements. Simulations using a mathematical model, parameterized with new Brazilian data, were conducted to compare and contrast projections of vector control achieved with the alternative approaches. Used as a standalone approach, both technologies have their disadvantages: RIDL suppression of the mosquito population is temporary and Wolbachia deployments may produce transient increases in resident mosquito populations. However, strategically combining both approaches resulted in mitigation of the risks of inadvertent exacerbation of the wild mosquito population and longer term control [85].

### Improving personal protection against Aedes mosquitoes

Although a number of promising arbovirus transmission-blocking/population-suppression technologies are currently in development for *Aedes* mosquitoes, such as *Wolbachia* [86] and sterile-male releases [87], there are a deficit of immediately implementable solutions. Personal protective technologies (PPT), such as repellent clothing, exist and show efficacy [88], but are seldom utilised on a large scale [89,90]. We conducted focus group interviews involving pregnant women in Colombia with the aim of identifying areas where PPT product design and information dissemination can be refined to encourage uptake. Using our findings, we are developing durable repellent formulations utilising cyclodextrin technologies that can be conveniently applied to clothing at home.

(II) **Research preparedness networks, capacity building, dissemination, and sustainability**

We have developed a platform called REDe (www.REDe.tghn.org) through the Global Health Network based at Oxford University (www.TGHN.org). This is a research capacity network and knowledge exchange platform that is a shared output from the three EU funded Zika consortia (ZikaPLAN, ZIKAction and ZIKAlliance). The aim is to create a strong network between all organisations and groups working on ZIKV and to use this as a mechanism to be ‘research ready’ for the next outbreak, as well as for disseminating outputs and recommendations from these studies in a highly accessible facility. Addressing the gap in research capacity in LMICs is pivotal in ensuring broad-based systems improvement, with local knowledge and training being central to responsive health system development [91]. Local solutions are also more likely to have buy-in from local providers and policymakers, and this ownership should result in solutions that are more sustainable than those imposed by others [91]. REDe means network in Portuguese and Spanish – and is a shared and open resource centre, where researchers and health workers can access tools, resources and support, enabling better and more research in the region. REDe operates both online and in the regions. Table 3 summarizes the initiatives further arising from ZikaPLAN. Within these regions, REDe has local coordinators embedded into research teams. Through this regional programme, there have been workshops and regional training efforts, which are supported through the online platform, thus strengthening the impact and need for sustainability. E-learning courses have also been developed. The facilities of REDe will be used to maximize the transfer of research into practice by summarizing the research output and by hosting the tools, resources, guidance and recommendations generated by our consortium. This will ensure better visibility and dissemination of our research methods and findings in addition to long-term knowledge transfer beyond the funding period. Table 3 summarizes some websites that post-ZikaPLAN created materials.

**Table 3.** Web-based information that ZikaPLAN has made publicly available.

Website	What is the website about!	URL
REDe	REDe is an international network focused on building research capacity and preparedness to tackle emerging infectious disease outbreaks in Latin America and Caribbean.	https://rede.tghn.org/
Global Birth Defects	The Global Birth Defects website is an initiative by the International Committee for Congenital Anomaly Surveillance Tools to provide specific and pragmatic resources that can improve surveillance systems and research projects in low-resource communities and areas where congenital anomaly diagnosis expertise is scarce.	https://globalbirthdefects.tghn.org/
Brain Infections Global	Improving the Management of Acute Brain Infections	https://braininfectionsglobal.tghn.org/
Global Vector Hub	The Global Vector Hub is an open access, interactive resource that not only has the capacity to transform vector research and vector control programmes, but also revolutionise our preparedness and ability to respond quickly and effectively to vector-borne disease outbreaks, around the world.	https://www.lshtm.ac.uk/research/centres-projects-groups/globalvectorhub
IGOS International Guillain-Barré syndrome outcome study	The study aims to identify clinical and biological determinants and predictors of disease course in Guillain-Barré syndrome.	https://gbsstudies.erasmusmc.nl/
IGOS-Zika	The International Zika virus related Guillain-Barré syndrome Outcome Study (IGOS-Zika)	https://igoszika.erasmusmc.nl/

To maximize our output, we built up a governance structure together with ZIKAlliance and ZIKAction with a reciprocal clinical monitoring platform, a joint quality assurance programme for the laboratory diagnostics, and established principles of governance and data sharing, all explicitly described in two joint work packages. We set up common bodies for the management of the scientific programme, including a common Scientific Advisory Board, a common Ethics Advisory Committee, a Communication Oversight Board, and cross-consortia working groups.

### Outlook

Our consortium will further capitalize on the platforms established and the experience gained through this urgent ZIKV research response, in order to evolve into *a network capable of rapidly launching a research response* to future severe infectious outbreaks caused by emerging pathogens with pandemic potential or potential to cause significant damage to health and socioeconomic wellbeing in the region. Our initial research platform will be further developed through a comprehensive ‘inter-epidemic’ action plan addressing and fine-tuning the response to any obstacles identified during the ZIKV research response (e.g. resolving regulatory and other bottlenecks, development of adaptable study protocols, strengthening ICT infrastructure for communication and information exchange, developing a training programme to enhance the local partners’ capacity for laboratory and clinical research, developing a communication strategy for patient and public engagement, etc.); as outlined in the original EU call for Zika research. A comprehensive data-management framework allowing the standardized collection, storage, analysis, and sharing of data is being implemented together with the other Zika consortia for our clinical cohort studies under the guidance of WHO [13]. Additionally, we are working towards a sustainability strategy and business plan that would enable the continuation of the network beyond the timeline of the EU grant. To this end, ZikaPLAN is now part of the European Clinical Research Alliance on Infectious Diseases (ECRAID: https://www.jpiamr.eu/kick-off-ecraid-the-european-clinical-research-alliance-on-infectious-diseases/), another EU funded consortium to pro-actively address the sustainability of infectious diseases networks to combat the impact of newly emerging pathogens and enhance epidemic preparedness. The project’s vision is to establish a coordinated and permanent infrastructure for research on arboviral diseases in the Americas. The vision is to become a self-sustaining, permanent, single access ‘go-to-network’ for arboviral diseases, with a common research agenda, organizational structures and processes, thereby harvesting synergies across the networks. Ongoing funding will be challenging. However, as important as the ad-hoc emergency driven EU funding was for Zika, it would be a shame if all the efforts and networks would dissolve after the ending of the 4-year project, thus preventing the sustained impact of the knowledge and infrastructures developed. If and when new arboviral outbreaks emerge such as Ross river [92], yellow fever [93], chikungunya [94] or Mayaro [95], new ad-hoc networks would need to be established with the inherent risk of inefficiencies, lack of synergies and predictable delays. Therefore, the EU and other funding agencies need to start thinking about funding mechanisms to ensure the sustained impact of the knowledge and infrastructures developed through the recently funded three Zika consortia, ZikaPLAN, ZIKAlliance and ZIKAction, in order to quickly respond to new arboviral outbreaks. True transparent research partnerships are needed between Europe and Latin America, and not just parachutists [96]. We need not only bilateral but global research partnerships that are based on trust, mutual benefit and sharing, and are able to respond rapidly leveraging upon already existing structures and networks.

## Paper context

Zika virus infection emerged as a public health emergency of international concern and the European Commission awarded grants to three research consortia. This paper synthesises mid-term achievements of ZikaPLAN, presenting findings that address the knowledge gaps to combat ZIKV and our efforts to build research capacity and a preparedness network in Latin American and Caribbean region to support, thus enabling sustained research in the region.

## References

[1] Heymann DL, Hodgson A, Sall AA, et al Zika virus and microcephaly: why is this situation a PHEIC? Lancet. 2016;387:719–16. 26876373 10.1016/S0140-6736(16)00320-2 PMC7134564

[2] Wilder-Smith A, Preet R, Renhorn KE, et al ZikaPLAN: Zika preparedness Latin American network. Glob Health Action. 2017;10:1398485. 29235414 10.1080/16549716.2017.1398485 PMC7011980

[3] Wilder-Smith A, Tissera H, AbuBakar S, et al Novel tools for the surveillance and control of dengue: findings by the DengueTools research consortium. Glob Health Action. 2018;11:1549930. 30560735 10.1080/16549716.2018.1549930 PMC6282436

[4] de Araujo TV, Rodrigues LC, de Alencar Ximenes RA, et al Association between Zika virus infection and microcephaly in Brazil, January to May, 2016: preliminary report of a case-control study. Lancet Infect Dis. 2016;16:1356–1363. 27641777 10.1016/S1473-3099(16)30318-8 PMC7617035

[5] Miranda-Filho DB, Martelli CM, Ximenes RA, et al Initial description of the presumed congenital Zika syndrome. Am J Public Health. 2016;106:598–600. 26959258 10.2105/AJPH.2016.303115 PMC4816005

[6] Paixao ES, Barreto F, Teixeira MG, et al History, epidemiology, and clinical manifestations of Zika: a systematic review. Am J Public Health. 2016;106:606–612. 26959260 10.2105/AJPH.2016.303112 PMC4816002

[7] Rodrigues LC. Zika: the tragedy and the opportunities. Am J Public Health. 2016;106:582. 26959248 10.2105/AJPH.2016.303114 PMC4816004

[8] Albuquerque MF, Souza WV, Mendes AD, et al Pyriproxyfen and the microcephaly epidemic in Brazil - an ecological approach to explore the hypothesis of their association. Mem Inst Oswaldo Cruz. 2016;111:774–776. 27812601 10.1590/0074-02760160291 PMC5146741

[9] Jacobs BC, van den Berg B, Verboon C, et al International Guillain-Barre syndrome outcome study: protocol of a prospective observational cohort study on clinical and biological predictors of disease course and outcome in Guillain-Barre syndrome. J Peripher Nerv Syst. 2017;22:68–76. 28406555 10.1111/jns.12209

[10] Whitehead SS Development of TV003/TV005, a single dose, highly immunogenic live attenuated dengue vaccine; what makes this vaccine different from the Sanofi-Pasteur CYD vaccine? Expert Rev Vaccines. 2016;15:509–517. 26559731 10.1586/14760584.2016.1115727 PMC4956407

[11] Song BH, Yun SI, Woolley M, et al Zika virus: history, epidemiology, transmission, and clinical presentation. J Neuroimmunol. 2017;308:50–64. 28285789 10.1016/j.jneuroim.2017.03.001

[12] Awadh A, Chughtai AA, Dyda A, et al Does Zika virus cause microcephaly - applying the Bradford Hill viewpoints. PLoS Curr. 2017;9. 10.1371/currents.outbreaks.2fced6e886074f6db162a00d4940133b PMC5346029 28357156

[13] Wilder-Smith A, Wei Y, Araujo TVB, et al Understanding the relation between Zika virus infection during pregnancy and adverse fetal, infant and child outcomes: a protocol for a systematic review and individual participant data meta-analysis of longitudinal studies of pregnant women and their infants and children. BMJ Open. 2019;9:e026092. 10.1136/bmjopen-2018-026092 PMC6588966 31217315

[14] de Araujo TVB, Ximenes RAA, Miranda-Filho DB, et al Association between microcephaly, Zika virus infection, and other risk factors in Brazil: final report of a case-control study. Lancet Infect Dis. 2018;18:328–336. 29242091 10.1016/S1473-3099(17)30727-2 PMC7617036

[15] Sheldrick RC, Perrin EC Evidence-based milestones for surveillance of cognitive, language, and motor development. Acad Pediatr. 2013;13:577–586. 24238685 10.1016/j.acap.2013.07.001 PMC3993976

[16] Prechtl HF, Einspieler C, Cioni G, et al An early marker for neurological deficits after perinatal brain lesions. Lancet. 1997;349:1361–1363. 9149699 10.1016/S0140-6736(96)10182-3

[17] Msall ME Measuring outcomes after extreme prematurity with the Bayley-III Scales of infant and toddler development: a cautionary tale from Australia. Arch Pediatr Adolesc Med. 2010;164:391–393. 20368495 10.1001/archpediatrics.2010.25

[18] Lopes Moreira ME, Nielsen-Saines K, Brasil P, et al Neurodevelopment in infants exposed to Zika virus in Utero. N Engl J Med. 2018;379:2377–2379. 30575464 10.1056/NEJMc1800098 PMC6478167

[19] Dos Santos SFM, Soares FVM, de Abranches AD, et al Infants with microcephaly due to ZIKA virus exposure: nutritional status and food practices. Nutr J. 2019;18:4. 30634976 10.1186/s12937-019-0429-3 PMC6330418

[20] Einspieler C, Utsch F, Brasil P, et al Association of infants exposed to prenatal Zika virus infection with their clinical, neurologic, and developmental status evaluated via the general movement assessment tool. JAMA Network Open. 2019;2:e187235. 30657537 10.1001/jamanetworkopen.2018.7235 PMC6431234

[21] Albuquerque MSV, Lyra TM, Melo APL, et al Access to healthcare for children with congenital Zika syndrome in Brazil: perspectives of mothers and health professionals. Health Policy Plan. 2019. 10.1093/heapol/czz059 PMC6788207 31369667

[22] Duttine A, Smythe T, Calheiro de Sa MR, et al Development and assessment of the feasibility of a Zika family support programme: a study protocol. Wellcome Open Res. 2019;4:80. 31289753 10.12688/wellcomeopenres.15085.1 PMC6600857

[23] Orioli IM, Dolk H, Lopez-Camelo JS, et al Prevalence and clinical profile of microcephaly in South America pre-Zika, 2005–14: prevalence and case-control study. BMJ. 2017;359:j5018. 29162597 10.1136/bmj.j5018 PMC5696624

[24] Mier YT-RL, Delorey MJ, Sejvar JJ, et al Guillain-Barre syndrome risk among individuals infected with Zika virus: a multi-country assessment. BMC Med. 2018;16:67. 29759069 10.1186/s12916-018-1052-4 PMC5952697

[25] Mehta R, Soares CN, Medialdea-Carrera R, et al The spectrum of neurological disease associated with Zika and chikungunya viruses in adults in Rio de Janeiro, Brazil: A case series. PLoS Negl Trop Dis. 2018;12:e0006212. 29432457 10.1371/journal.pntd.0006212 PMC5837186

[26] Cao-Lormeau VM, Blake A, Mons S, et al Guillain-Barre Syndrome outbreak associated with Zika virus infection in French polynesia: a case-control study. Lancet. 2016;387:1531–1539. 26948433 10.1016/S0140-6736(16)00562-6 PMC5444521

[27] Parra B, Lizarazo J, Jimenez-Arango JA, et al Guillain-Barre syndrome associated with Zika virus infection in Colombia. N Engl J Med. 2016;375:1513–1523. 27705091 10.1056/NEJMoa1605564

[28] Leonhard SE, Lant S, Jacobs BC, et al Zika virus infection in the returning traveller: what every neurologist should know. Pract Neurol. 2018;18:271–277. 29618586 10.1136/practneurol-2017-001789 PMC6204932

[29] Cumberworth SL, Barrie JA, Cunningham ME, et al Zika virus tropism and interactions in myelinating neural cell cultures: CNS cells and myelin are preferentially affected. Acta Neuropathol Commun. 2017;5:50. 28645311 10.1186/s40478-017-0450-8 PMC5481922

[30] Fischer C, Drosten C, Drexler JF The difficulties in obtaining reliable Zika virus diagnostics. Lancet Infect Dis. 2019;19:240–241. 30833056 10.1016/S1473-3099(19)30049-0

[31] Goncalves A, Peeling RW, Chu MC, et al Innovative and new approaches to laboratory diagnosis of Zika and dengue: a meeting report. J Infect Dis. 2018;217:1060–1068. 29294035 10.1093/infdis/jix678 PMC6279137

[32] Xu X, Vaughan K, Weiskopf D, et al Identifying candidate targets of immune responses in Zika virus based on homology to epitopes in other flavivirus species. PLoS Curr. 2016;8. 10.1371/currents.outbreaks.9aa2e1fb61b0f632f58a098773008c4b PMC5145810 28018746

[33] Grifoni A, Pham J, Sidney J, et al Prior dengue virus exposure shapes T cell immunity to Zika virus in humans. J Virol. 2017;91. 10.1128/JVI.01469-17 PMC5709580 28978707

[34] Ricciardi MJ, Magnani DM, Grifoni A, et al Ontogeny of the B- and T-cell response in a primary Zika virus infection of a dengue-naive individual during the 2016 outbreak in Miami, FL. PLoS Negl Trop Dis. 2017;11:e0006000. 29267278 10.1371/journal.pntd.0006000 PMC5755934

[35] Grifoni A, Costa-Ramos P, Pham J, et al Cutting edge: transcriptional profiling reveals multifunctional and cytotoxic antiviral responses of Zika virus-specific CD8(+) T cells. J Immunol. 2018;201:3487–3491. 30413672 10.4049/jimmunol.1801090 PMC6287102

[36] Metz SW, Gallichotte EN, Brackbill A, et al In vitro assembly and stabilization of dengue and Zika virus envelope protein homo-dimers. Sci Rep. 2017;7:4524. 28674411 10.1038/s41598-017-04767-6 PMC5495877

[37] Premkumar L, Collins M, Graham S, et al Development of envelope protein antigens to serologically differentiate Zika virus infection from dengue virus infection. J Clin Microbiol. 2018;56. 10.1128/JCM.01504-17 PMC5824056 29263206

[38] Montoya M, Collins M, Dejnirattisai W, et al Longitudinal analysis of antibody cross-neutralization following Zika virus and dengue virus infection in Asia and the Americas. J Infect Dis. 2018;218:536–545. 29618091 10.1093/infdis/jiy164 PMC6047418

[39] Pettersson JH, Eldholm V, Seligman SJ, et al How did Zika virus emerge in the Pacific Islands and Latin America? MBio. 2016;7. 10.1128/mBio.01239-16 PMC5061869 27729507

[40] Falconar AK, Martinez F The NS1 glycoprotein can generate dramatic antibody-enhanced dengue viral replication in normal out-bred mice resulting in lethal multi-organ disease. PLoS One. 2011;6:e21024. 21731643 10.1371/journal.pone.0021024 PMC3120820

[41] Pettersson JH, Bohlin J, Dupont-Rouzeyrol M, et al Re-visiting the evolution, dispersal and epidemiology of Zika virus in Asia. Emerg Microbes Infect. 2018;7:79. 29739925 10.1038/s41426-018-0082-5 PMC5940881

[42] van Boheemen S, Tas A, Anvar SY, et al Quasispecies composition and evolution of a typical Zika virus clinical isolate from Suriname. Sci Rep. 2017;7:2368. 28539654 10.1038/s41598-017-02652-w PMC5443807

[43] Mead PS, Duggal NK, Hook SA, et al Zika virus shedding in semen of symptomatic infected men. N Engl J Med. 2018;378:1377–1385. 29641964 10.1056/NEJMoa1711038

[44] Yakob L, Kucharski A, Hue S, et al Low risk of a sexually-transmitted Zika virus outbreak. Lancet Infect Dis. 2016;16:1100–1102. 27676337 10.1016/S1473-3099(16)30324-3

[45] Faria NR, Quick J, Claro IM, et al Establishment and cryptic transmission of Zika virus in Brazil and the Americas. Nature. 2017;546:406–410. 28538727 10.1038/nature22401 PMC5722632

[46] Massad E, Burattini MN, Khan K, et al On the origin and timing of Zika virus introduction in Brazil. Epidemiol Infect. 2017;1–10. 10.1017/S0950268817001200 PMC9148813 28675351

[47] O’Reilly KM, Lowe R, Edmunds WJ, et al Projecting the end of the Zika virus epidemic in Latin America: a modelling analysis. BMC Med. 2018;16:180. 30285863 10.1186/s12916-018-1158-8 PMC6169075

[48] Duong V, Dussart P, Buchy P Zika virus in Asia. Int J Infect Dis. 2017;54:121–128. 27939768 10.1016/j.ijid.2016.11.420

[49] Watts AG, Huber C, Bogoch II, et al Potential Zika virus spread within and beyond India. J Travel Med. 2018;25. 10.1093/jtm/tay132 30476232

[50] Hamer DH, Chen LH Zika in Angola and India. J Travel Med. 2019;26. 10.1093/jtm/taz012 30753689

[51] Massad E, Coutinho FAB, Wilder-Smith A Modelling an optimum vaccination strategy against ZIKA virus for outbreak use. Epidemiol Infect. 2019;147:e196. 31364534 10.1017/S0950268819000712 PMC6536754

[52] Glaesser D, Kester J, Paulose H, et al Global travel patterns: an overview. J Travel Med. 2017;24. 10.1093/jtm/tax007 28637267

[53] Leder K, Grobusch MP, Gautret P, et al Zika beyond the Americas: travelers as sentinels of Zika virus transmission. A GeoSentinel analysis, 2012 to 2016. PLoS One. 2017;12:e0185689. 28973011 10.1371/journal.pone.0185689 PMC5626466

[54] Wilder-Smith A, Chang CR, Leong WY Zika in travellers 1947–2017: a systematic review. J Travel Med. 2018;25. 10.1093/jtm/tay044 30016469

[55] Wang G, Zheng W, Zhu S, et al A cluster of Zika virus infection among travellers returning to China from Samoa: a case tracing study. J Travel Med. 2018;25. 10.1093/jtm/tay022 29718403

[56] Zhang J, Jin X, Zhu Z, et al Early detection of Zika virus infection among travellers from areas of ongoing transmission in China. J Travel Med. 2016;23:taw047. 10.1093/jtm/taw047 27378370

[57] Summers DJ, Acosta RW, Acosta AM Zika virus in an American recreational traveler. J Travel Med. 2015;22:338–340. 25996909 10.1111/jtm.12208

[58] Sokal A, D’Ortenzio E, Houhou-Fidouh N, et al Zika virus infection: report of the first imported cases in a Paris travel centre. J Travel Med. 2016;24:taw066. 10.1093/jtm/taw066 28679155

[59] Shinohara K, Kutsuna S, Takasaki T, et al Zika fever imported from Thailand to Japan, and diagnosed by PCR in the urines. J Travel Med. 2016;23:tav011. 10.1093/jtm/tav011 26782128

[60] Katanami Y, Kutsuna S, Taniguchi S, et al Detection of Zika virus in a traveller from Vietnam to Japan. J Travel Med. 2017;24. 10.1093/jtm/tax031 28498965

[61] Torresi J, Steffen R Redefining priorities towards graded travel-related infectious disease research. J Travel Med. 2017;24. 10.1093/jtm/tax064 29088486

[62] Leder K, Steffen R, Cramer JP, et al Risk assessment in travel medicine: how to obtain, interpret, and use risk data for informing pre-travel advice. J Travel Med. 2015;22:13–20. 25378126 10.1111/jtm.12170

[63] Wilder-Smith A, Boggild AK Sentinel surveillance in travel medicine: 20 years of GeoSentinel publications (1999–2018). J Travel Med. 2018;25. 10.1093/jtm/tay139 30508133

[64] Chen LH, Leder K, Barbre KA, et al Business travel-associated illness: a GeoSentinel analysis. J Travel Med. 2018;25. 10.1093/jtm/tax097 PMC5824651 29462444

[65] Boggild AK, Geduld J, Libman M, et al Spectrum of illness in migrants to Canada: sentinel surveillance through CanTravNet. J Travel Med. 2019;26. 10.1093/jtm/tay117 30395252

[66] Angelo KM, Haulman NJ, Terry AC, et al Illness among US resident student travellers after return to the USA: a GeoSentinel analysis, 2007–17. J Travel Med. 2018;25. 10.1093/jtm/tay074 PMC6503850 30202952

[67] Boggild AK, Esposito DH, Kozarsky PE, et al Differential diagnosis of illness in travelers arriving from Sierra Leone, Liberia, or Guinea: a cross-sectional study from the GeoSentinel surveillance network. Ann Intern Med. 2015;162:757–764. 25961811 10.7326/M15-0074 PMC4629254

[68] Michal Stevens A, Esposito DH, Stoney RJ, et al Clostridium difficile infection in returning travellers. J Travel Med. 2017;24. 10.1093/jtm/taw099 PMC5697897 28355613

[69] Salzer HJF, Stoney RJ, Angelo KM, et al Epidemiological aspects of travel-related systemic endemic mycoses: a GeoSentinel analysis, 1997–2017. J Travel Med. 2018;25. 10.1093/jtm/tay055 PMC6628256 30085265

[70] Barbosa F, Barnett ED, Gautret P, et al Bordetella pertussis infections in travelers: data from the GeoSentinel global network. J Travel Med. 2017;24. 10.1093/jtm/taw094 28355615

[71] Neumayr A, Munoz J, Schunk M, et al Sentinel surveillance of imported dengue via travellers to Europe 2012 to 2014: TropNet data from the DengueTools research initiative. Euro Surveill. 2017;22. 10.2807/1560-7917.ES.2017.22.1.30433 PMC5388098 28080959

[72] Huits R, Van den Bossche D, Eggermont K, et al Incidence of Zika virus infection in a prospective cohort of Belgian travellers to the Americas in 2016. Int J Infect Dis. 2019;78:39–43. 30368020 10.1016/j.ijid.2018.10.010

[73] Huits R, Maniewski U, Van den Bossche D, et al A cross-sectional analysis of Zika virus infection in symptomatic and asymptomatic non-pregnant travellers: experience of a European reference center during the outbreak in the Americas. Travel Med Infect Dis. 2019;27:107–114. 30205195 10.1016/j.tmaid.2018.08.007

[74] Wilder-Smith A Can dengue virus be sexually transmitted? J Travel Med. 2019;26. 10.1093/jtm/tay157 30590715

[75] Molton JS, Low I, Choy MMJ, et al Dengue virus not detected in human semen. J Travel Med. 2018;25. 10.1093/jtm/tay023 29672710

[76] Huits R, De Smet B, Arien KK, et al Zika virus in semen: a prospective cohort study of symptomatic travellers returning to Belgium. Bull World Health Organ. 2017;95:802–809. 29200521 10.2471/BLT.17.181370 PMC5710082

[77] Chen LH, Hamer DH Zika virus and sexual transmission: updated preconception guidance. J Travel Med. 2018;25. 10.1093/jtm/tay095 30289482

[78] Quam MB, Wilder-Smith A Estimated global exportations of Zika virus infections via travellers from Brazil from 2014 to 2015. J Travel Med. 2016;23:taw059. 10.1093/jtm/taw059 27601533

[79] Tuite AR, Thomas-Bachli A, Acosta H, et al Infectious disease implications of large-scale migration of Venezuelan nationals. J Travel Med. 2018;25. 10.1093/jtm/tay077 PMC6142906 30192972

[80] Massad E, Tan SH, Khan K, et al Estimated Zika virus importations to Europe by travellers from Brazil. Glob Health Action. 2016;9:31669. 27193266 10.3402/gha.v9.31669 PMC4871896

[81] Furuya-Kanamori L, Liang S, Milinovich G, et al Co-distribution and co-infection of chikungunya and dengue viruses. BMC Infect Dis. 2016;16:84. 26936191 10.1186/s12879-016-1417-2 PMC4776349

[82] Yakob L, Walker T Zika virus outbreak in the Americas: the need for novel mosquito control methods. Lancet Glob Health. 2016;4:e148–e9. 26848089 10.1016/S2214-109X(16)00048-6

[83] Ritchie SA Wolbachia and the near cessation of dengue outbreaks in Northern Australia despite continued dengue importations via travellers. J Travel Med. 2018;25. 10.1093/jtm/tay084 30247733

[84] Black WCT, Alphey L, James AA Why RIDL is not SIT. Trends Parasitol. 2011;27:362–370. 21659002 10.1016/j.pt.2011.04.004

[85] Yakob L, Funk S, Camacho A, et al Aedes aegypti control through modernized, integrated vector management. PLoS Curr. 2017;9. 10.1371/currents.outbreaks.45deb8e03a438c4d088afb4fafae8747 PMC5319873 28286698

[86] Sinkins SP Wolbachia and arbovirus inhibition in mosquitoes. Future Microbiol. 2013;8:1249–1256. 24059916 10.2217/fmb.13.95

[87] Harris AF, McKemey AR, Nimmo D, et al Successful suppression of a field mosquito population by sustained release of engineered male mosquitoes. Nat Biotechnol. 2012;30:828–830. 22965050 10.1038/nbt.2350

[88] Schreck CE, Haile DG, Kline DL The effectiveness of permethrin and deet, alone or in combination, for protection against Aedes taeniorhynchus. Am J Trop Med Hyg. 1984;33:725–730. 6148024 10.4269/ajtmh.1984.33.725

[89] Lalani T, Yun H, Tribble D, et al A comparison of compliance rates with anti-vectorial protective measures during travel to regions with dengue or chikungunya activity, and regions endemic for Plasmodium falciparum malaria. J Travel Med. 2016;23:taw043. 10.1093/jtm/taw043 PMC4939934 27378367

[90] Goodyer L, Schofield S Mosquito repellents for the traveller: does picaridin provide longer protection than DEET? J Travel Med. 2018;25:S10–S5. 29718433 10.1093/jtm/tay005

[91] Beran D, Byass P, Gbakima A, et al Research capacity building-obligations for global health partners. Lancet Glob Health. 2017;5:e567–e8. 28495256 10.1016/S2214-109X(17)30180-8

[92] Shanks GD Could Ross River virus be the next Zika? J Travel Med. 2019;26. 10.1093/jtm/taz003 30657934

[93] Gubler DJ Pandemic yellow fever: a potential threat to global health via travelers. J Travel Med. 2018;25. 10.1093/jtm/tay097 30299494

[94] Rezza G Chikungunya is back in Italy: 2007–2017. J Travel Med. 2018;25. 10.1093/jtm/tay004 29669058

[95] Acosta-Ampudia Y, Monsalve DM, Rodriguez Y, et al Mayaro: an emerging viral threat? Emerg Microbes Infect. 2018;7:163. 30254258 10.1038/s41426-018-0163-5 PMC6156602

[96] Heymann DL, Liu J, Lillywhite L Partnerships, not parachutists, for Zika research. N Engl J Med. 2016;374:1504–1505. 26958936 10.1056/NEJMp1602278

